# Risk model based on genes regulating the response of tumor cells to T-cell-mediated killing in esophageal squamous cell carcinoma

**DOI:** 10.18632/aging.205495

**Published:** 2024-02-01

**Authors:** Xun Zhang, Chuting Yu, Siwei Zhou, Yanhui Zhang, Bo Tian, Yan Bian, Wei Wang, Han Lin, Luo-Wei Wang

**Affiliations:** 1 Department of Gastroenterology, Changhai Hospital, Naval Medical University, Shanghai, China; 2 National Clinical Research Center for Digestive Diseases, Shanghai, China

**Keywords:** esophageal squamous cell carcinoma, T-cell-mediated tumor killing, tumor microenvironment, immunotherapy, risk score

## Abstract

Immune checkpoint inhibitors (ICIs) represent a promising therapeutic approach for esophageal squamous cell carcinoma (ESCC). However, the subpopulations of ESCC patients expected to benefit from ICIs have not been clearly defined. The anti-tumor cytotoxic activity of T cells is an important pharmacological mechanism of ICIs. In this study, the prognostic value of the genes regulating tumor cells to T cell-mediated killing (referred to as GRTTKs) in ESCC was explored by using a comprehensive bioinformatics approach. Training and validation datasets were obtained from The Cancer Genome Atlas (TCGA) and Gene Expression Omnibus (GEO), respectively. A prognostic risk scoring model was developed by integrating prognostic GRTTKs from TCGA and GEO datasets using a ridge regression algorithm. Patients with ESCC were divided into high- and low-risk groups based on eight GRTTKs (*EIF4H*, *CDK2*, *TCEA1*, *SPTLC2*, *TMEM209*, *RGP1*, *EIF3D*, and *CAPZA3*) to predict overall survival in the TCGA cohort. Using Kaplan-Meier curves, receiver operating characteristic curves, and C-index analysis, the high reliability of the prognostic risk-scoring model was certified. The model scores served as independent prognostic factors, and combining clinical staging with risk scoring improved the predictive value. Patients in the high-risk group exhibited abundant immune cell infiltration, including immune checkpoint expression, antigen presentation capability, immune cycle gene expression, and high tumor inflammation signature scores. The high-risk group exhibited a greater response to immunotherapy and neoadjuvant chemotherapy than the low-risk group. Drug sensitivity analysis demonstrated lower IC50 for AZD6244 and PD.0332991 in high-risk groups and lower IC50 for cisplatin, ATRA, QS11, and vinorelbine in the low-risk group. Furthermore, the differential expression of GRTTK-related signatures including CDK2, TCEA1, and TMEM209 were verified in ESCC tissues and paracancerous tissues. Overall, the novel GRTTK-based prognostic model can serve as indicators to predict the survival status and immunotherapy response of patients with ESCC, thereby providing guidance for the development of personalized treatment strategies.

## INTRODUCTION

Esophageal cancer, a type of malignant gastrointestinal cancer, ranked seventh in incidence and sixth in mortality in 2020 worldwide [1]. Esophageal squamous cell carcinoma (ESCC) and esophageal adenocarcinoma are the two main histopathological subtypes [2]. ESCC accounts for approximately 90% of esophageal cancers, with the highest incidence rates in Eastern Asia and Eastern Africa [3]. Owing to the lack of obvious symptoms and specific diagnostic biomarkers in the early stages of ESCC, patients are often diagnosed in the late stages [4], and the 5-year overall survival rate of ESCC is still less than 30% [5]. For clinical management, a comprehensive, multidisciplinary treatment model involving surgery, radiotherapy, chemotherapy, targeted therapy, and immunotherapy is a promising approach to ESCC [6]. Recent developments in immunotherapy that harness the patient’s immune system, have shown encouraging therapeutic effects in various types of tumors [7–9]. For example, the protein PD-1, which negatively regulates T lymphocytes, binds to its ligands PD-L1 and PD-L2, resulting in the suppression of lymphocyte activation and inhibiting the immune response. Anti-PD1 and anti-PD-L1 antibodies disrupt the interaction of PD1 with PD-L1/PD-L2, thereby reactivating T lymphocyte immune function and exerting antitumor effects [10]. As PD-L1 expression is enriched on the surface of tumor cells in patients with ESCC [11], Immune checkpoint inhibitors (ICIs) directed at PD-1 and its counterpart PD-L1 have displayed encouraging outcomes in the management of advanced ESCC. For instance, pembrolizumab resulted in longer overall survival (OS) than chemotherapy alone, leading to its approval as a second-line treatment for advanced ESCC [12]. Other checkpoint inhibitors, such as nivolumab and camrelizumab, have also shown efficacy [13, 14]. Despite recent advances in immunotherapy for ESCC, such as immune checkpoint inhibitors (ICIs) targeting the PD-1/PD-L1 axis, identifying patients who may benefit remains a challenge [15, 16]. With advances in high-throughput sequencing technology and bioinformatic methods, numerous studies have explored biomarkers to predict the immune status and prognosis of patients with ESCC. For example, Guo et al. developed a six-gene prognostic signature correlated with m6A RNA methylation regulators to predict PD-L1 expression and immune cell infiltration in ESCC [17]. Angiogenesis gene panels have also been used to predict ESCC prognosis and immunotherapy [18]. However, these prognostic models lack effectiveness in clinical applications, and more practical prognostic indicators are required to guide precise immunotherapy for ESCC.

T lymphocytes have antigen-specific cytotoxic capabilities and are central to the activation of the immune system against cancer [19]. Dysfunctions in T-cell function limit the efficacy of tumor immunotherapy [20]. T-cell-mediated tumor killing (TTK) is a major principle of ICI therapy. Pan et al. utilized a genome-scale screen to identify genes associated with resistance to T-cell-mediated killing, including *Pbrm1*, *Arid2*, and *Brd7* in a chromatin remodeling complex in melanoma cells. Inactivation of *Pbrm1* increased the sensitivity of tumor cells to interferon-γ and made tumor cells more susceptible to T-cell-mediated killing [21]. Kishton et al. applied multi-omics approaches to identify several genes, such as *RAF2*, *BIRC2*, and *ALG11*, capable of limiting T-cell killing activity, and demonstrated that knocking down *BIRC2* using CRISPR Cas9 technology can effectively enhance the efficacy of immunotherapy [22]. Prognostic models of T-cell-mediated killing-related genes in hepatocellular carcinoma and lung adenocarcinoma have been explored [23, 24]. However, to the best of our knowledge, studies on genes that regulate the response of tumor cells to T-cell-mediated tumor killing (termed GRTTKs) in ESCC are lacking.

In the present research, we explored the prognostic relevance and immunological significance of GRTTKs in patients with ESCC. A prognostic model based on GRTTKs was established using data from The Cancer Genome Atlas (TCGA) for risk stratification and prognostic prediction. Moreover, we conducted an analysis of prognosis and the tumor immune microenvironment disparities between the high-risk and low-risk groups. Clinical treatment and sensitivity to different chemotherapy and immunotherapy drugs were also evaluated. The findings of this study indicate a significant correlation between T-cell-mediated tumor cell killing and the tumor microenvironment in ESCC, providing a basis for clinical decision-making in patients with ESCC.

## RESULTS

### Characteristics of GRTTKs in ESCC

**Figure 1:**
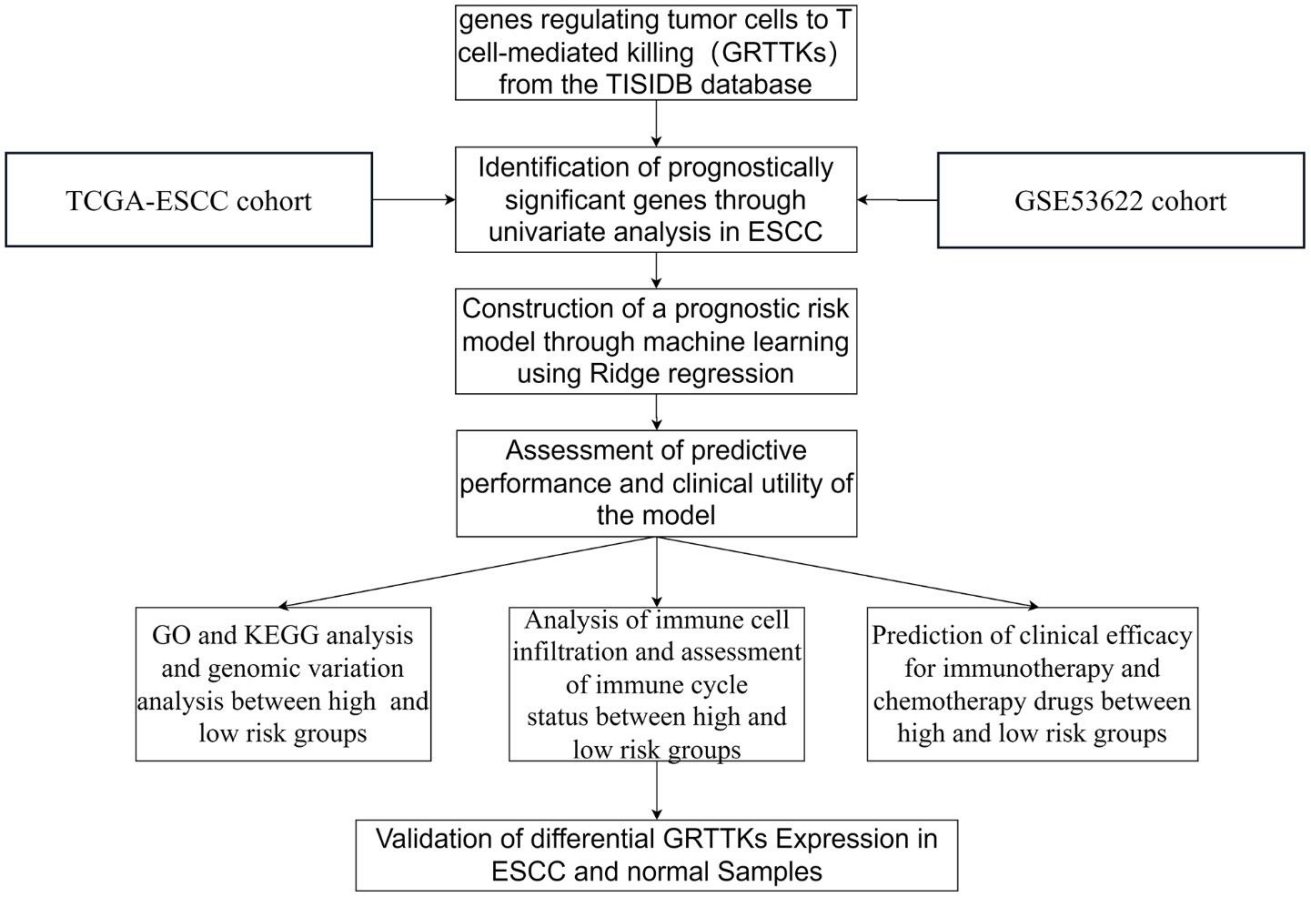
Flow chat of this study.

**Figure 2:**
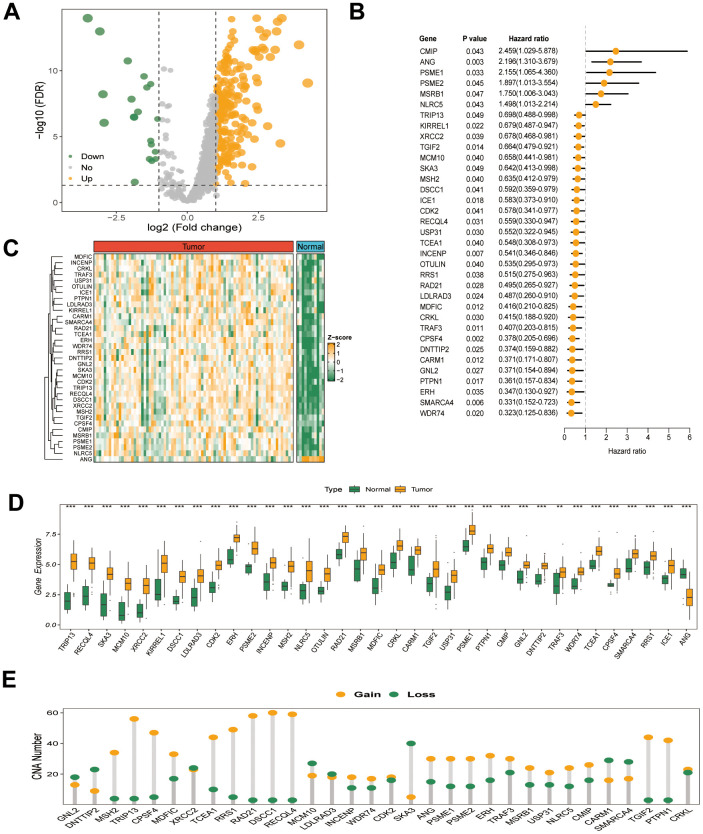
(**A**) Volcano plot of the differential expression of 270 GRTTKs between tumor and normal samples in the TCGA-ESCC cohort. (**B**) Univariate Cox regression analysis of 35 GRTTKs and prognosis in ESCC. (**C**) Heatmap displayed displaying the differential expression of 35 GRTTKs between tumor and normal samples. (**D**) Boxplots of the differential expression of 35 GRTTKs between tumor and normal samples. (**E**) Copy number variation (CNV) of 35 GRTTKs in ESCC.

The study’s workflow is illustrated in Figure 1. Transcriptome expression profiles for ESCC tissues and normal esophageal samples were obtained from TCGA and GSE53622 cohorts. Subsequently, comprehensive bioinformatic analysis was conducted on the collected data. The multidimensional omics data of GRTTKs were analyzed using the TCGA-ESCC cohort. Comparing tumor samples with normal samples in the TCGA-ESCC cohort revealed 250 up-regulated and 20 down-regulated GRTTKs, as shown in the volcano plot in Figure 2A. Univariate Cox regression analyses indicated that 35 of the 270 GRTTKs were associated with ESCC prognosis. Six were identified as risk factors (HR > 1) and twenty-nine as protective factors (HR < 1) for ESCC prognosis (Figure 2B). Comparisons of the expression levels of the 35 GSTTKs between tumor and normal samples are shown in heatmaps and box plots in Figure 2C, 2D. Additionally, we investigated the copy number variation (CNV) within these 35 GRTTKs. A significant increase in copy number was observed for *RAD21, DSCC1*, and *RECQL4*, whereas notable losses were detected for *DNTTIP2*, *SKA3*, *XRCC2*, and *MCM10* in ESCC tissues compared with those of the healthy controls (Figure 2E). We further analyzed the mutation landscape of the 35 GSTTKs using a waterfall plot. In the TCGA-ESCC cohort, we observed a predominance of missense mutations and a high frequency of single nucleotide polymorphisms (SNPs), with TP53 exhibiting the most significant somatic mutations (Supplementary Figure 1A). Four GRTTKs, including *SMARCA4*, *NLRC5*, *ICE1*, and *USP31* exhibited mutations with frequencies exceeding 1% (Supplementary Figure 1B).

**Figure 3:**
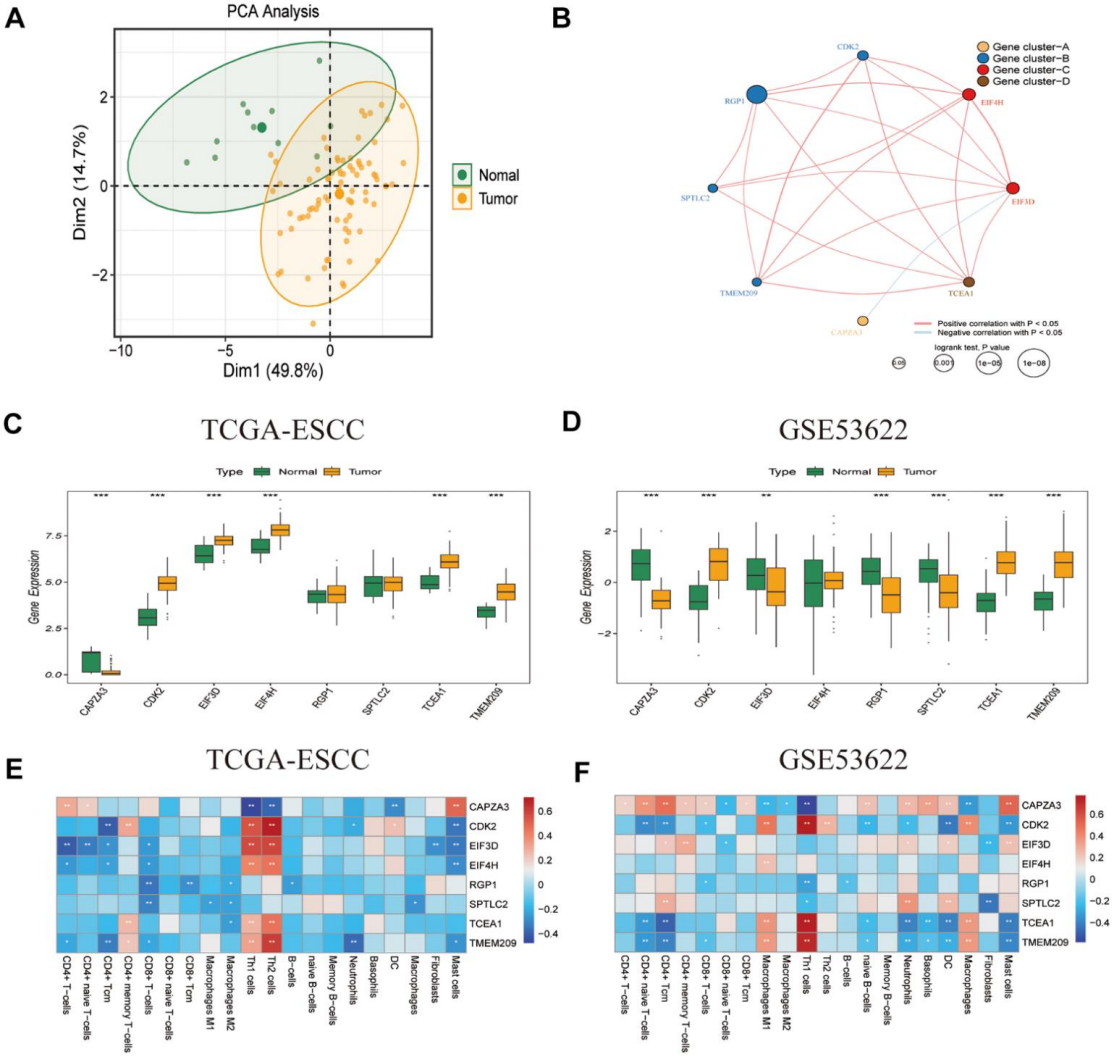
**Characteristics of prognostic GRTTK in TCGA-ESCC and GSE53622.** (**A**) PCA separated tumor samples from normal samples based on GRTTK. (**B**) The correlation network revealed internal connections among the eight prognostic GRTTKs. (**C**, **D**) Box plots depicting the differential expression between tumor and normal samples among the eight genes in TCGA-ESCC and GSE53622. (**E**, **F**) Pearson’s correlation coefficients between the expression of the eight genes and various immune cells in TCGA-ESCC and GSE53622.

To screen for genes with consistent prognostic significance in the heterogeneous cohort, we applied the criteria of P < 0.1 and HR > 1 or P < 0.1 and HR < 1 in both TCGA-ESCC and GSE53622 datasets. Eight GRTTKs with prognostic significance (*CAPZA3*, *CDK2*, *EIF3D*, *EIF4H*, *RGP1*, *SPTLC2*, *TCEA1*, and *TMEM209*) in both cohorts were identified (Supplementary Table 1). PCA revealed the capacity of the eight prognostic GRTTKs to differentiate between tumor and normal groups within the TCGA-ESCC cohort (Figure 3A). Interactions among the eight GRTTKs were explored using clinical information and transcriptomic features from the TCGA-ESCC cohort, revealing four distinct patterns (Figure 3B). The expression differences for these eight genes between tumor and normal samples in both TCGA-ESCC and GSE53622 cohorts are displayed using boxplots in Figures 3C, 3D. Subsequently, the associations between TIICs and the prognostic GRTTKs were evaluated. *CDK2*, *EIF3D*, *EIF4H*, *TCEA1*, and *TMEM209* demonstrated substantial positive correlations with Th1 and Th2 cells in the TCGA-ESCC cohort (Figure 3E). In the GSE53622 cohort, the expression levels of *CDK2*, *TCEA1*, and *TMEM209* were significantly negatively correlated with CD4+ Tcm, neutrophils, dendritic cells, and mast cells (Figure 3F). These findings indicate that GRTTK expression not only differs between tumors and normal samples but also has a robust prognostic value, demonstrating a strong association with the tumor microenvironment.

### Construction of a risk model based on prognostic GRTTKs

**Figure 4:**
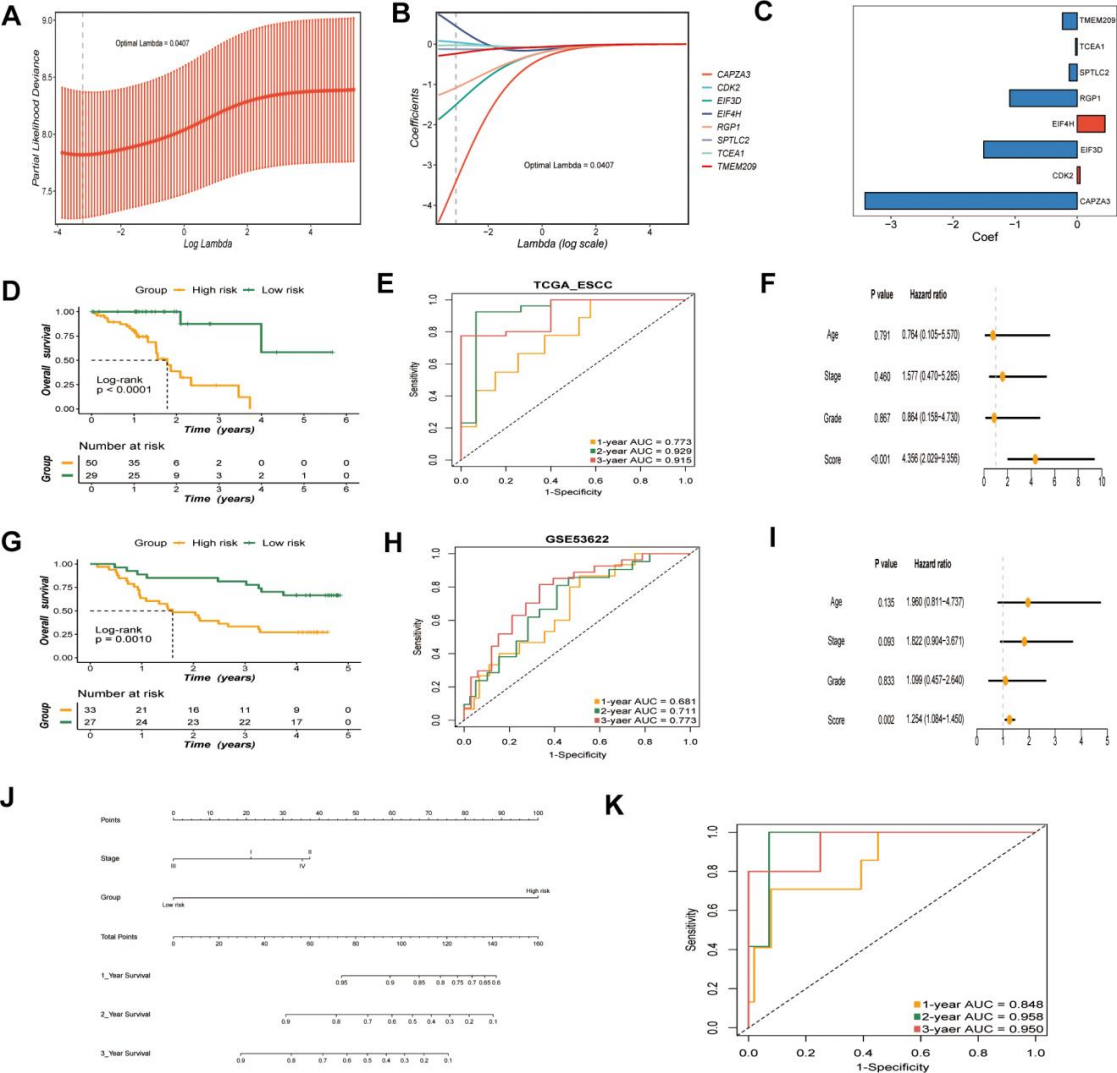
**Construction and validation of the GRTTK based risk model.** (**A**) Optimal regularization parameter (λ) selection (λ = 0.0407) using minimum partial likelihood deviance for TCGA cohort. (**B**) Coefficients of the risk model corresponding to the optimal λ. (**C**) Coefficient values for the eight genes in the risk model. (**D**) Kaplan-Meier analysis of overall survival (OS) based on risk groups in TCGA cohort. (**E**) ROC curves evaluating risk model performance in TCGA cohort. (**F**) Multivariable Cox analysis of the TCGA-ESCC cohort. (**G**) OS in the high-risk and low-risk groups in the GEO cohort (P = 0.0010). (**H**) ROC curves for the risk model for the GSE53622 cohort. (**I**) Multivariate Cox analysis of the GSE53622 cohort. (**J**) A nomogram displaying the predictive value of clinical stage and risk models. (**K**) AUC for the combined evaluation of patient prognosis using clinical stage and risk scores.

A risk model was established with prognostic GRTTKs using ridge regression based on optimal lambda (λ = 0.0407) and matching coefficient values. The risk score was determined as follows: (EIF4H*0.4472) + (CDK2*0.0463) - (TCEA1*0.0262) -(SPTLC2*0.1268) - (TMEM209*0.2362) - (RGP1*1.0850) - (EIF3D*1.5052) - (CAPZA3*3.4184) (Figure 4A–4C). According to the prognostic model, we calculated the scores for each patient in the TCGA-ESCC dataset; patients in the low-risk group had significantly longer survival times than those in the high-risk group (P < 0.0001) (Figure 4D). UMAP revealed the distribution of samples belonging to high- and low-risk groups in TCGA-ESCC. (Supplementary Figure 2A). The risk-scoring system exhibited favorable predictive capability, with corresponding AUC of 0.773, 0.929, and 0.915 for 1-year, 2-year, and 3-year survival, respectively (Figure 4E). We further incorporated age, clinical stage, pathological grade, and other variables into a multivariate Cox regression analysis, demonstrating that risk score was an independent prognostic factor for OS (Figure 4F).

GSE53622 was used as an external dataset for further validation. Kaplan-Meier survival analysis yielded similar results in the GSE53622 validation cohort (P = 0.0010) (Figure 4G). The sample distributions of different groups were differentiated by the UMAP algorithm in the GSE53622 dataset (Supplementary Figure 2B). The AUC values for the GSE53622 cohort at 1, 2, and 3-years were 0.681, 0.711, and 0.773, respectively (Figure 4H). Multivariate Cox regression analysis of the GSE53622 validation set identified the prognostic model score as an independent prognostic factor (Figure 4I). Moreover, the C index of the model for the TCGA-ESCC and GSE53622 cohorts were 0.783 and 0.670, respectively, demonstrating the stability of the model. (Supplementary Figure 2C). In summary, our risk model exhibited high predictive accuracy.

To further explore the association between the model and clinical stage, we analyzed the correlation between the risk model and clinical stage. A nomogram was plotted to visualize the predictive value of incorporating both clinical stage and risk scores. (Figure 4J). The AUC of combining clinical stage and risk score was calculated by time-ROC analysis in TCGA-ESCC cohort. The resulting AUC values for 1-, 2-, and 3-year overall survival were 0.848, 0.958, and 0.950, respectively (Figure 4K).

### Functions and somatic mutations associated with GRTTKs patterns

**Figure 5:**
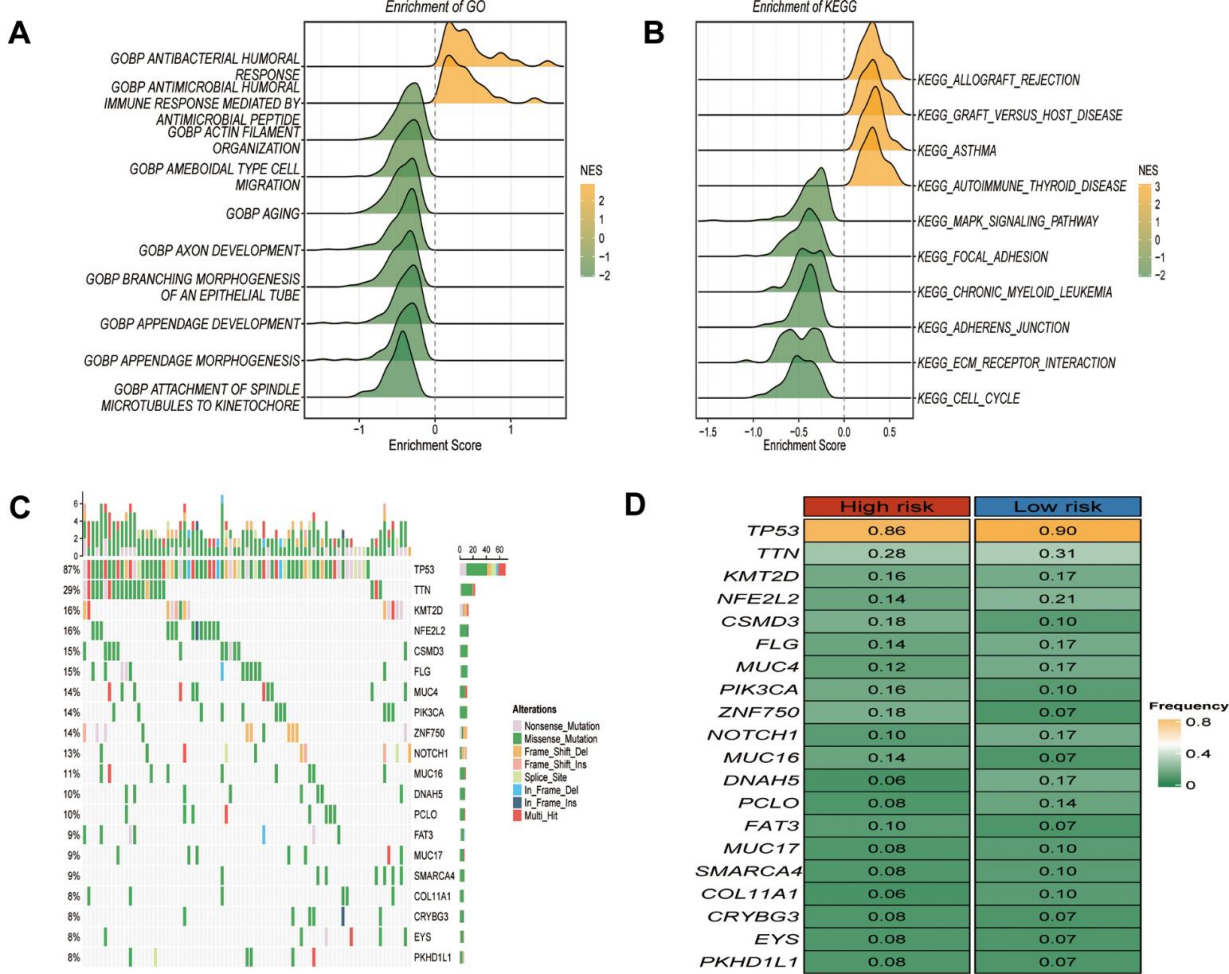
**Functional enrichment and somatic mutations related to GRTTK patterns.** (**A**, **B**) GO and KEGG enrichment analysis of differentially expressed genes between high and low risk group. (**C**) Waterfall plot illustrating the overall mutation landscape in ESCC. (**D**) Graphical representation contrasting somatic mutation frequencies between high and low risk groups.

Functional enrichment analysis was constructed to characterize the genes in the risk model. GO analysis suggested that GSTTKs were enriched in functions related to immune-related responses, such as the antibacterial humoral response and antimicrobial humoral immune response mediated by antimicrobial peptides (Figure 5A). KEGG enrichment analysis showed that these genes were mainly involved in allograft-rejection, the MAPK -signaling pathway, and the cell cycle (Figure 5B). In the GRTTK model, *TP53* demonstrated the highest discrepancy in mutation frequency between the groups (Figure 5C, 5D).

### Tumor immune microenvironment and status in different GRTTK Groups

**Figure 6:**
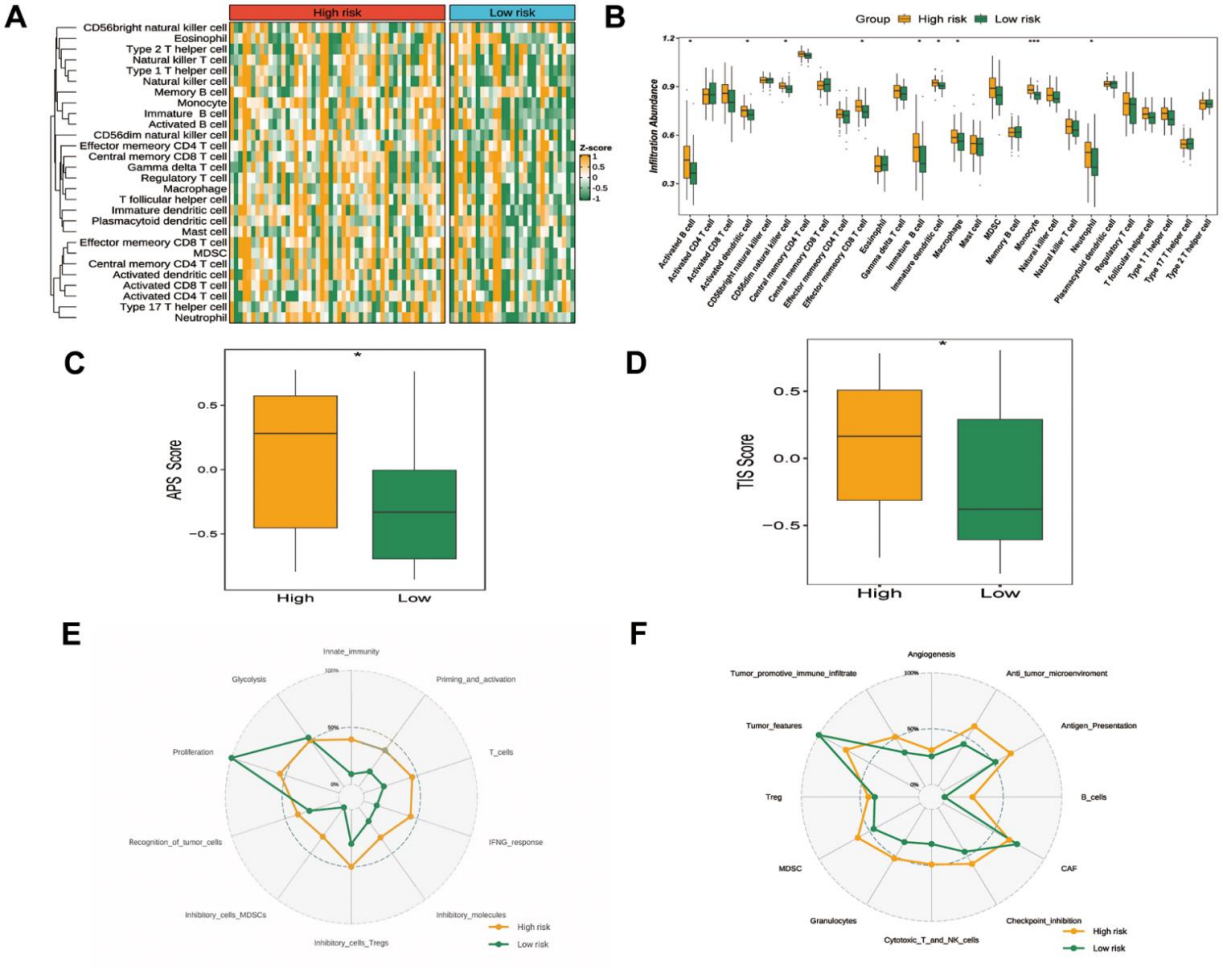
**Tumor immune microenvironment and status in different GRTTK groups.** (**A**) A heatmap exhibiting ssGSEA enrichment scores for 28 immunogenic cell type markers, stratified by high- and low-risk groups as per the GRTTK prognostic model. (**B**) Relative abundance of infiltrating cell types differed across different risk groups based on GRTTKs. (**C**) Assessment and contrast of antigen presentation potential between high- and low-risk groups, stratified using the GRTTK prognostic signatures. (**D**) Contrast of TIS scores between high-risk and low-risk patient groups. (**E**, **F**) Radar chart depicting divergence in immune cycle gene set enrichment between risk groups based on the GRTTK model.

We further examined the association between immunotherapy and immune characteristics. Heatmap analysis revealed higher immune cell infiltration in the high-risk versus low-risk group (Figure 6A). Significantly more activated B cells, dendritic cells, natural killer cells, effector T cells, immature B/dendritic cells, macrophages, monocytes and neutrophils were observed in the high-risk group (Figure 6B). The high-risk group also exhibited higher antigen presentation score (Figure 6C) and tumor inflammation signature score (Figure 6D), indicating greater responsiveness to ICIs. We also investigated the features of the immune cycle within the tumor microenvironment [25, 26]. The immunocycle gene set was evaluated. As illustrated using a radar plot, the high-risk group showed significantly enhanced immune reserves, whereas the low-risk group displayed more pronounced tumor phenotypes (Figure 6E, 6F). Moreover, in an analysis of expression differences of various immune checkpoint molecules including 27 co-stimulatory factors, 15 co-inhibitory factors, and 9 antigen-presenting factors, the high-risk group showed higher expression of immune checkpoint molecules *CD48* and *HLA-DPB1*. (Supplementary Figure 3A–3C).

### Relationship between drug sensitivity and the GSTTK risk model

**Figure 7:**
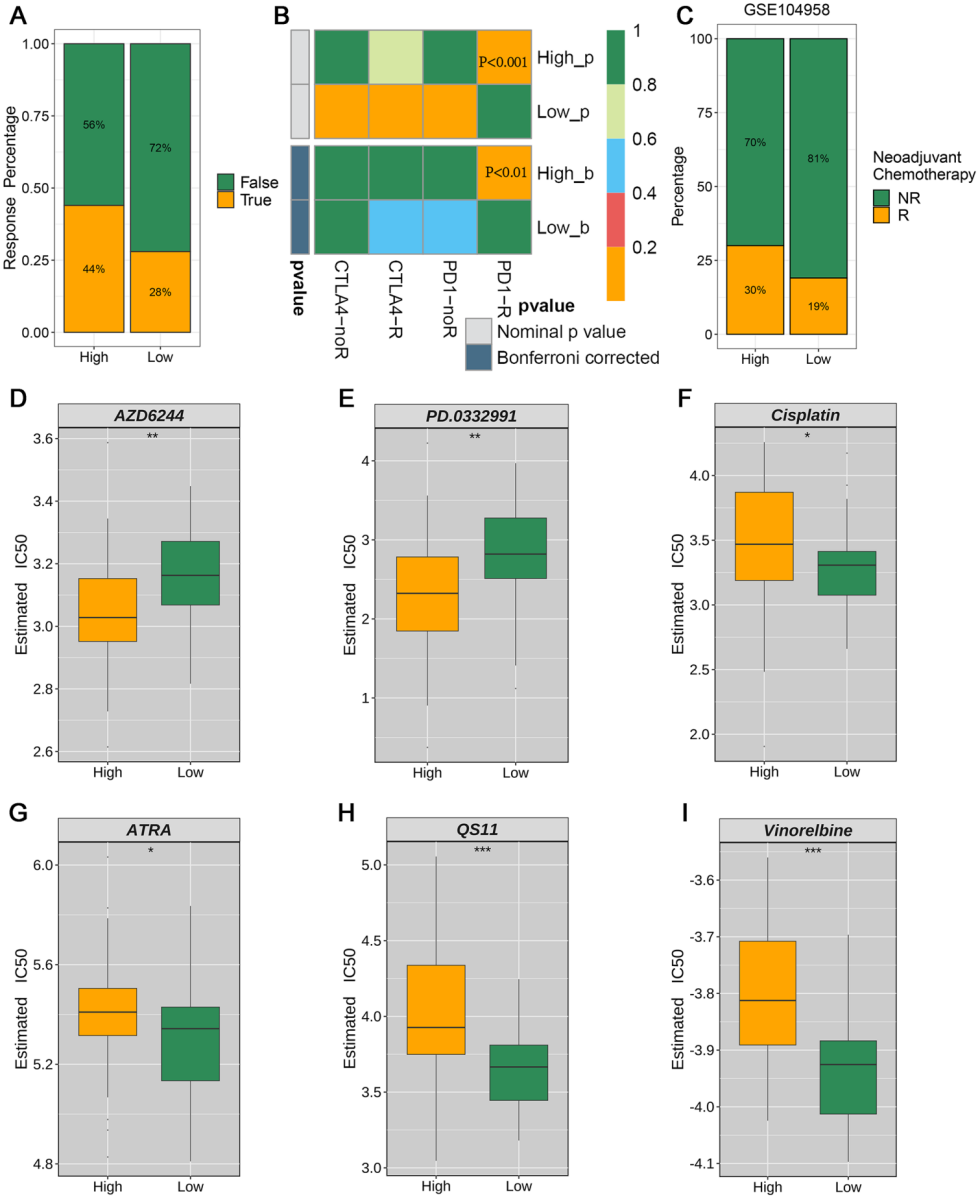
Evaluation of clinical immunotherapy responses and drug sensitivity (**A**) TIDE analysis forecasts the proportion of responsive patients to immunotherapy in the high- and low-risk groups stratified by the prognostic model. (**B**) SubMap analysis unveiled significant divergence in anti-PD-1 immunotherapy response between the high-risk and low-risk groups. (**C**) Proportions of neoadjuvant chemotherapy response between high-risk and low-risk patient groups in the GSE104958 data set. (**D**–**I**) Treatment response rates to six chemotherapy agents based on drug sensitivity scores.

To further explore the clinical utility of the newly developed signature, we compared immunotherapy and chemotherapy responses and drug sensitivity between the risk groups. Therapeutic response to ICIs was evaluated and predicted using the TIDE website. The high-risk group showed a 44% immunotherapy response rate versus 28% for the low-risk group using the TIDE prediction (Figure 7A). Similarly, SubMap analysis revealed a consistent trend in the response to immunotherapy among patients with ESCC, consistent with the results of the TIDE database analysis (Figure 7B). The high-risk group showed a significantly higher response rate (30% vs. 19%) than the low-risk group, as evidenced by analyzing of the GSE104958 neoadjuvant chemotherapy cohort (Figure 7C). The high-risk group showed sensitivity to AZD6244 and PD.0332991 (Figure 7D, 7E), whereas the low-risk group demonstrated potential sensitivity to cisplatin, ATRA, QS11, and vinorelbine (Figure 7F–7I).

### Verification of differential GRTTK expression in ESCC tissue samples

**Figure 8:**
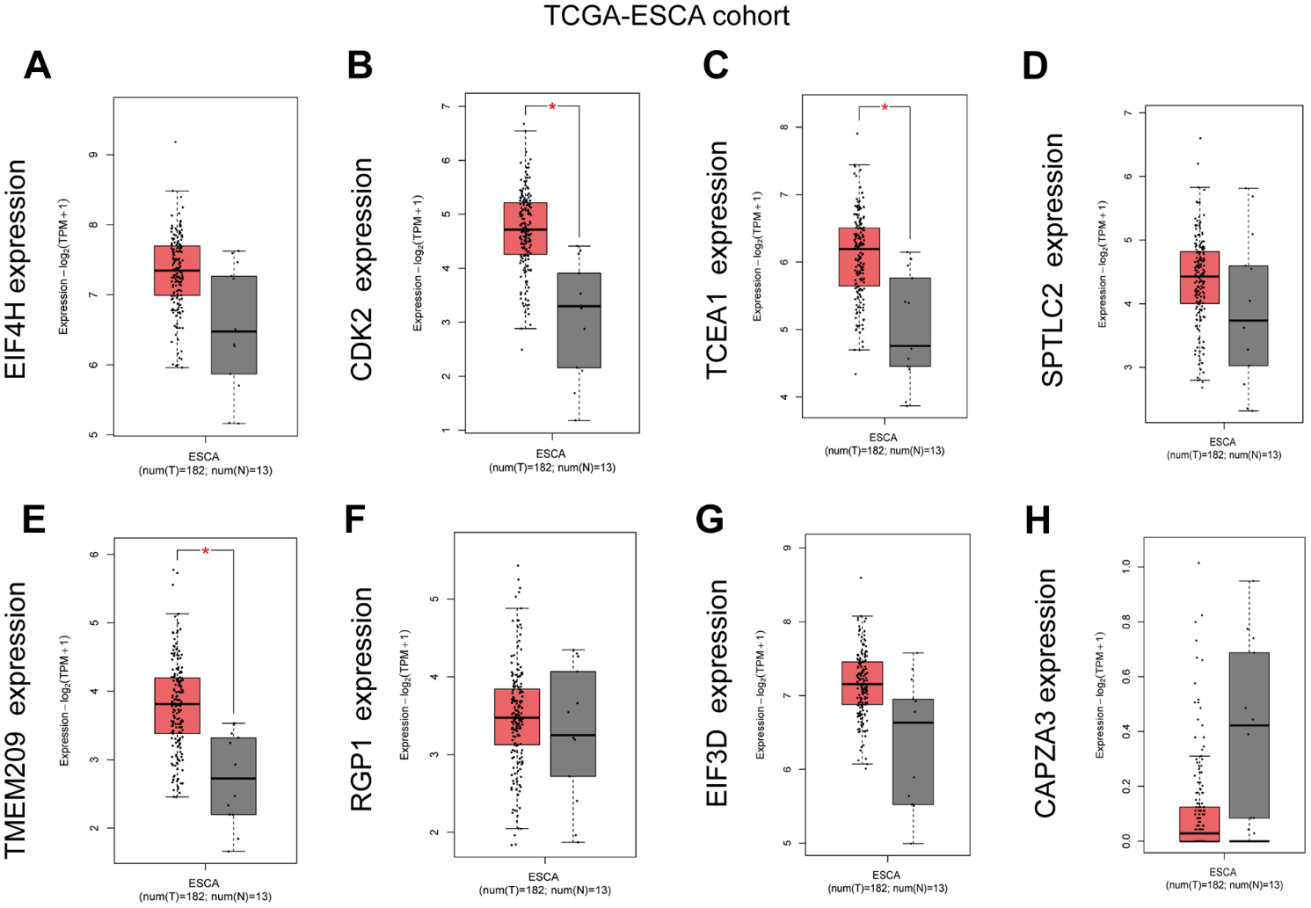
**Expression Level of GRTTKs in GEPIA database.** (**A**–**H**) The GEPIA database was utilized to contrast mRNA expression levels of pivotal genes between ESCC and normal tissues.

**Figure 9:**
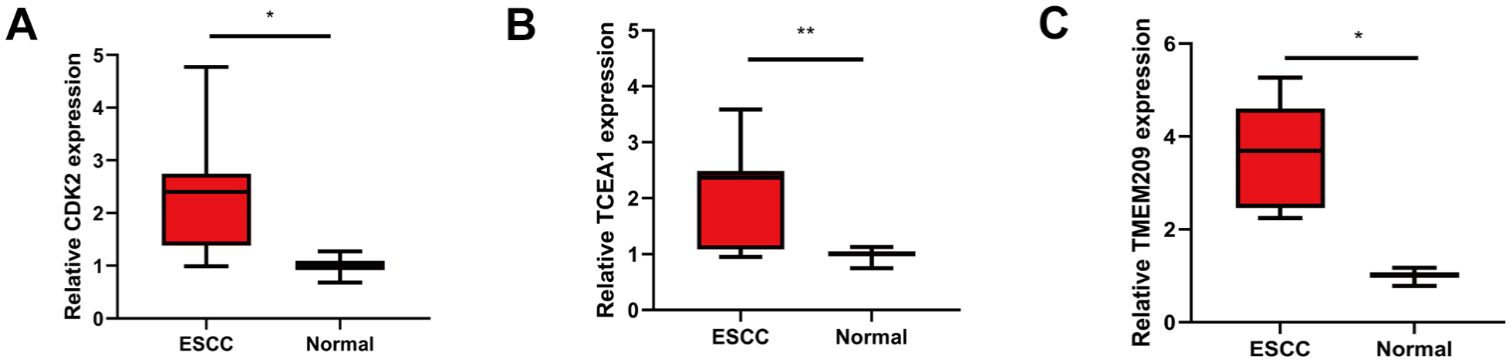
**Validation the differential expression of GRTTK in ESCC tissues.** (**A**–**C**) Differential expression levels of CDK2, TCEA1 and TMEM209 in ESCC and normal tissues.

We used GEPIA database to analyze the expression of eight GRTTKs in TCGA-ESCA cohort (Figure 8A–8H). The expression levels of *CDK2*, *TCEA1* and *TMEM209* in the TCGA-ESCA cohort were upregulated in ESCC tissues compared with those in normal tissues. Consequently, the mRNA expression analysis was conducted in patients with ESCC and adjacent normal tissue samples using qRT-PCR to elevate the levels of *CDK2*, *TCEA1*, and *TMEM209* genes. The results revealed an upregulation in the expression of *CDK2*, *TCEA1*, and *TMEM209* within the primary tumorous tissues of ESCC patients in comparison to the adjacent non-cancerous tissues (Figure 9A–9C). These results are consistent with the outcomes of the bioinformatic analysis, reinforcing the validity of the experimental results.

## DISCUSSION

Surgery combined with radiotherapy and chemotherapy is one of the primary treatment approaches for advanced ESCC [27]. In recent years, ICIs have significantly changed the treatment landscape and demonstrated clinical efficacy in advanced esophageal cancer, including ESCC [13, 28]. However, owing to tumor heterogeneity and the complexity of carcinogenic mechanisms, immunotherapy benefits only a subset of patients with ESCC [29], which poses a challenge in the application of this approach. An expanding number of researches show that the immune response within the tumor microenvironment affects tumor development, prognosis, and anti-tumor immunity [30]. A crucial role for cancer immunotherapy is T-cell-mediated immunotoxicity. Enhancing the sensitivity of cancer cells to T-cell-mediated killing is a key strategy for alleviating immune resistance. Hence, prognostic evaluations based on GRTTK patterns are crucial to facilitate precision-oriented clinical treatment.

In this study, we identified eight GRTTKs associated with the development and prognosis of ESCC including *EIF4H*, *CDK2*, *TCEA1*, *SPTLC2*, *TMEM209*, *RGP1*, *CAPZA3* and *EIF3D* based on the transcriptional matrix profiling analysis of TCGA and GEO cohorts. Eukaryotic Translation Initiation Factor 4H(*EIF4H*) is a pivotal gene involved in the initiation of protein synthesis in eukaryotic organisms [31]. It generates two distinct isoforms through alternative splicing processes and is implicated in the progression of lung adenocarcinoma [32]. *CDK2* in tumors plays a pivotal role in cell cycle regulation and proliferation control, potentially serving as a promising therapeutic target for cancer treatment. Zhou et al. reported the overexpression of *CDK2* gene in ESCC, which is consistent with our analytical findings, implicating its involvement in tumor proliferation [33]. *TCEA1*, a regulator of gene transcription, has been shown to influence myeloid cell proliferation and differentiation [34]. *SPTLC2* encodes serine palmitoyltransferase long chain base subunit 2, which is involved in the synthesis of neuronal sphingolipids. Mutations in this gene have been shown to inhibit the response of human T cells [35]. *TMEM209*, which encodes a nuclear envelope protein, has been reported to be associated with lung cancer [36]. *GRP1*, also referred to as *TIF32*, constitutes a component of the eukaryotic translation initiation factor 3 (eIF3) complex; it is localized within the cytoplasm and actively participates in the activity of translation initiation factors [37]. *EIF3D* is a subfamily member of the eukaryotic translation initiation factor 3 (eIF3), has been observed in various human cancers, implicating its involvement in tumorigenesis [38]. The *CAPZA3* gene encodes a protein associated with cellular cytoskeletal reorganization [39]. Although further research is needed to fully understand these prognostic genes in ESCC, our analysis underscores their significance as crucial prognostic factors. Moreover, these genes may serve as viable targets for treatment strategies. A significant difference in prognosis between risk groups is a prerequisite for group stratification [40]. A higher risk group had a worse prognosis compared to a low-risk group in this study. Prognostic risk models for different subgroups provide a basis for clinical translation [41]. Regarding the correlation between the risk model and clinical stage, a nomogram revealed that the combined utilization of both indicators resulted in a higher predictive performance (as determined by the ROC curve) than that of either parameter alone. This suggests that the prognostic model developed in this study can serve as a complementary tool for clinicopathological staging and improve prognostic prediction in clinical settings.

We developed a scoring system and constructed a prognostic model to evaluate the tumor immune microenvironment in high- and low-risk groups (such as tumor-infiltrating cell abundance, immune cell molecular markers, and immune regulatory gene expression) and explored the clinical and predictive significance of the immunotherapy response. Moreover, we assessed genetic variations, including somatic mutations, mutation features, and relevant signaling pathways, to investigate the etiological factors that drive GRTTK patterns.

Tumor microenvironments contain both cellular and non-cellular components, including immune cells, fibroblasts, endothelial cells, and extracellular matrix components, which contribute to the occurrence and development of tumors [42]. These components interact with tumor cells and, regulate the immune responses in the tumor microenvironment [43]. We performed ssGSEA, immune checkpoint molecule expression, and antigen presentation scoring to further investigate the differences in immune function between the high- and low-risk groups. The high- and low-risk groups demonstrated differences in monocytes, CD8+ T cells, macrophages, and neutrophils. The difference in monocyte immune cells was the most significant. Monocytes exert a profound effect on the tumor microenvironment via multiple mechanisms, leading to antitumor effects and activation of antigen-presenting cells [44]. Accurate prediction of antigen presentation represents a critical step in determining the ability of new antigens to activate antigen-specific T cells and effectively eliminate tumor cells [45]. Potentially, checkpoint inhibitors targeting PD-1, PD-L1 and CTLA-4 could counteract immunosuppressive cells populations that dominate the tumor microenvironment in esophageal squamous cell carcinoma. By blocking these inhibitory receptors on T cells, these agents may enhance the activation, proliferation and cytotoxic functions of tumor-reactive CD8+ T cells. Likewise, certain checkpoint inhibitors could stimulate immunogenic cell death, thereby increasing antigen presentation and tumor-specific T cell recognition. Additionally, the T -cell inflamed gene-expression profile serves as a potential indicator of the response and clinical efficacy of ICI therapy in cancer [46]. In this study, the high-risk groups demonstrated elevated APS and TIS scores. By integrating the evaluations of these immunological scoring-related indicators, we determined that patients in the high-risk group had a higher level of immune reserves, which may indicate a higher potential for response to immunotherapy. There is evidence that regulatory T cells (Tregs), tolerogenic dendritic cells, alternatively activated macrophages dominate the immune microenvironment of ESCC, resulting in immunosuppression. Targeting these cells to reactivate the anti-tumor immune response in ESCC may partly explain why the high-risk groups may respond better to immunotherapy [47].

Furthermore, we used the TIDE database to forecast the proportion of immunotherapy-responsive patients. The high-risk group exhibited significantly higher response rates, which was further supported by a SubMap analysis, suggesting that patients in the high-risk group may benefit more from immunotherapy. Accordingly, the integration of immunotherapy into the treatment regimen is recommended. Recently, a novel neoadjuvant chemotherapy regimen consisting of docetaxel, cisplatin, and 5-fluorouracil was investigated, demonstrating a high response rate for the treatment of advanced ESCC [48]. Based on our analysis, high-risk groups responded better to neoadjuvant chemotherapy than low-risk groups. The high-risk group showed sensitivity to AZD6244, also known as selumetinib, a MEK inhibitor [49], and PD.0332991, a highly specific, small- molecule inhibitor of CDK4 and 6 [50], providing a basis for the development of novel therapeutic strategies.

Although our study provides valuable insights, it has certain limitations, and a larger cohort of clinical samples is needed for validation. The GRTTK model and risk scores were derived using a comprehensive bioinformatics analysis. Accordingly, key GRTTKs must be validated and further evaluated by functional assays. Future studies should consider alternative bioinformatics methods or emerging technologies, such as machine learning or artificial intelligence, to improve the accuracy and robustness of the model.

In conclusion, we developed a prognostic model based on GRTTKs and performed risk stratification, providing insights into the role of T- cell activity in ESCC. Furthermore, we investigated biological functions, immune infiltration, immune status, and therapeutic efficacy associated with GRTTK risk stratification. These findings are valuable for guiding treatment strategies, such as the selection of immunotherapy or combined approaches. Ultimately, the results of this study enhance our understanding of the genomic characteristics of T-cell-related genes in ESCC.

## MATERIALS AND METHODS

### Publicly available data collection and processing

We obtained gene expression profiles along with associated clinical data including 80 tumor samples and 11 normal samples from the UCSC-Xena database (https://xenabrowser.net/datapages/) [51] and used as the training dataset. The GSE53622 cohort comprising 60 tumor samples and 60 normal samples was downloaded from The Gene Expression Omnibus (GEO) (https://www.ncbi.nlm.nih.gov/geo/) and utilized as the validation set. Mutation data for 80 ESCC tumor samples were obtained from the TCGA (https://portal.gdc.cancer.gov/), and the overall mutation landscape was evaluated using the plotmaf Summary function in the maftools R package. The Copy number variation data for TCGA-ESCC samples were retrieved from the UCSC-Xena database. The GSE104958 cohort consisted of ESCC samples and five normal samples from patients who received neoadjuvant treatment with docetaxel, cisplatin, and 5-fluorouracil, including gene expression profiles and corresponding treatment information. Raw data were normalized and transformed into log2(TPM + 1) format. A total of 1310 genes regulating tumor cell response to T cell-mediated killing, serving as GRTTKs, were retrieved from the TISIDB database [52] (http://cis.hku.hk/TISIDB/) (Supplementary Table 2). The expression levels of GRTTKs were examined between cancerous and adjacent normal tissues using GEPIA (Gene Expression Profiling Interactive Analysis, http://gepia2.cancer-pku.cn/#index), a web tool for differential gene expression analysis in cancer.

### Multi-omics analysis of GRTTKs in ESCC

The limma package was employed to identify differentially expressed GRTTKs within the TCGA-ESCC cohort, utilizing the following selection criteria: |log Fold Change (FC)| > 1 and False Discovery Rate (FDR) less than 0.05 [53]. The association of GRTTK with prognosis in ESCC was evaluated using univariate Cox regression analysis implemented in the Survival in package in R package. Principal Component Analysis (PCA) were utilized to assess the distribution of tumor and normal samples. Single-Sample Gene Set Enrichment Analysis (ssGSEA) were used to quantify GRTTK scores within the TCGA-ESCC samples. The infiltration scores for the TCGA-ESCC cohort were assessed using the ssGSEA algorithm in the GSVA R package based on specific immune cell markers [54]. The relationship between the immune, stromal, and ESTIMATE scores and GRTTK scores was determined based on Spearman correlation coefficients. The maftools package was employed for the characterization of somatic mutations within GRTTK-related genes in ESCC patients.

### Construction of a risk model related to GRTTK by ridge regression

Ridge regression, a biased estimation method useful for the analysis of collinear data, was used to construct a prognostic model based on GSTTK [55, 56]. Owing to the small sample size of some cohorts, consensus prognostic genes were ultimately filtered by univariate Cox analysis with thresholds of P < 0.1 and all hazard ratios (HR) of >1 or <1 for the TCGA-ESCC and GSE53622 cohorts [57]. The lambda.min function was used for optimization, which automatically selects the lambda value that leads to the smallest error in cross-validation. The prognostic score for each patient was calculated according to the gene expression level and the corresponding coefficient as follows:


$$\begin{array}{l} \text{Prognostic}\;\text{Score}\\ =\sum_{i=1}^{n}\text{Expression}\;(\text{i})\;\text{X}\;\text{Coefficient}\;(\text{i}) \end{array}$$

n indicates the overall number of genes in the signature, Expression(i) is the expression level of the gene, and Coefficient(i) is the regression coefficient for that gene. Patients within the TCGA-ESCC cohort were categorized into high-risk and low-risk groups based on their prognostic scores, with the median risk score serving as the dividing threshold. An analysis of survival rates in high-risk and low-risk groups was conducted with the “survival” R package, which used Kaplan-Meier survival curves to evaluate the differences between the groups. The predictive performance was evaluated using the concordance index (C-index) and receiver operating characteristic (ROC) -curve. The model was validated by using samples from the GSE53622 cohort. Patients in the GSE53622 cohort were scored using the same formula as the training cohort.

### Assessment of the efficacy and clinical value of the model

The effectiveness of the model as a clinical stratification tool for distinguishing between patients was evaluated using the UMAP algorithm. The area under the ROC curve (AUC) was used to calculate the accuracy of the model’s prognostic prediction. An analysis of multivariate Cox regression including age, clinical stage, and pathological grade was conducted to identify candidate predictive factors associated with survival (P < 0.05). Clinical calibration plots were constructed using the root-mean-squarer R package.

### Gene ontology (GO) and Kyoto Encyclopedia of Genes and Genomes (KEGG) analyses

In order to determine the ranked order of differentially expressed genes between the high-risk group and the low-risk group, log2FoldChange (log2FC) values were calculated using the limma R package. Following that, the clusterProfiler R package was used to identify KEGG and GO pathways associated with the risk groups, and the enriched pathways were visualized using the normalized enrichment score (NES). An adjusted P-value of 0.05 was used to filter functional candidates.

### Evaluation of the tumor microenvironment and immune cell subpopulations

An algorithm developed in the “estimate” package in R was used to calculate the stromal, immune, and ESTIMATE scores. In high- and low-risk groups, Wilcoxon tests were used to compare tumor-infiltrating immune cells (TIICs) proportionally. Based on the 28 characteristic marker genes of immune cells reported in a previous study [54], the ssGSEA algorithm in the GSVA R package [58] was used to obtain infiltration scores for immune cell subpopulations in different groups in the TCGA-ESCC cohort. Simultaneously, the expression levels of immune checkpoint molecules, including co-stimulatory [59], co-inhibitory [60] and antigen-presenting molecules [61], were quantified to assess the response to immune therapy in different risk groups. Tumor inflammation signature (TIS) analysis [62] was performed to determine the potential response of patients with ESCC to ICIs treatment. Furthermore, the immune cycle status of the different risk groups was evaluated using immune cycle gene sets [25].

### Immunotherapy and chemotherapy response evaluation

In high-risk and low-risk groups, the Tumor Immune Dysfunction and Exclusion (TIDE) website (http://tide.dfci.harvard.edu/) was used to predict ICIs responsiveness [63]. Similarities in mRNA expression patterns between patients with ESCC receiving immunotherapy, including treatment responders and non-responders, were assessed using SubMap. (https://cloud.genepattern.org/) [64]. The responses to neoadjuvant treatment in the high- and low-risk groups were assessed using the GSE104958 cohort [48]. The half-maximal inhibitory concentrations (IC50) of commonly employed chemotherapeutic drugs were calculated with the R package “pRRophetic” [65], which signifies the effectiveness of a substance in inhibiting specific biological or metabolic processes.

### ESCC tissue specimens

ESCC tumor tissues and corresponding adjacent normal tissues were collected from 12 patients at the First Affiliated Hospital of Naval Medical University between November 2021 and February 2022.

### RNA extraction and RT-PCR

A total RNA extraction was performed using TRIzol Reagent (Invitrogen, USA), according to the manufacturer’ s instructions. Following this, RNA was converted to cDNA using the HiScript II First Strand cDNA Synthesis Kit with gDNA wiper and qRT-PCR was performed using Taq Pro Universal SYBR qPCR Master Mix(Vazyme Biotech, Co., Ltd., Nanjing, China, R211-01). The design and synthesis of all primers were carried out by Shanghai Sangon Biotech and Listed in Supplementary Table 3. Subsequent analysis was conducted using the 2^–ΔΔCT^ method, with GAPDH serving as the internal reference.

### Statistical analysis

This study used R 4.1.2 to analyze and process the data, as well as generate plots. Spearman correlation analysis was used to perform the correlation analysis. Wilcoxon rank-sum tests or Student’s t-tests was used to analyze continuous variables. Categorical variables were analyzed using the chi-square test or Fisher’s exact test. Survival R package was used for Kaplan-Meier survival analysis and multivariate Cox regression analysis. Comparisons of survival rates were conducted using log-rank tests. Statistical significance was determined by two-tailed P-values with P <0.05 considered significant.

## Supplementary Material

Supplementary Figures

Supplementary Table 1

Supplementary Table 2

Supplementary Table 3
