# Supplementary material for: Risk model based on genes regulating the response of tumor cells to T-cell-mediated killing in esophageal squamous cell carcinoma
Source: Aging (Albany NY). 2024 Feb 1;16(3):2494–516. doi: 10.18632/aging.205495 (PMC10911339; doi:10.18632/aging.205495)
Supplement: Supplementary Figures [file aging-16-205495-s001.pdf]

SUPPLEMENTARY FIGURES

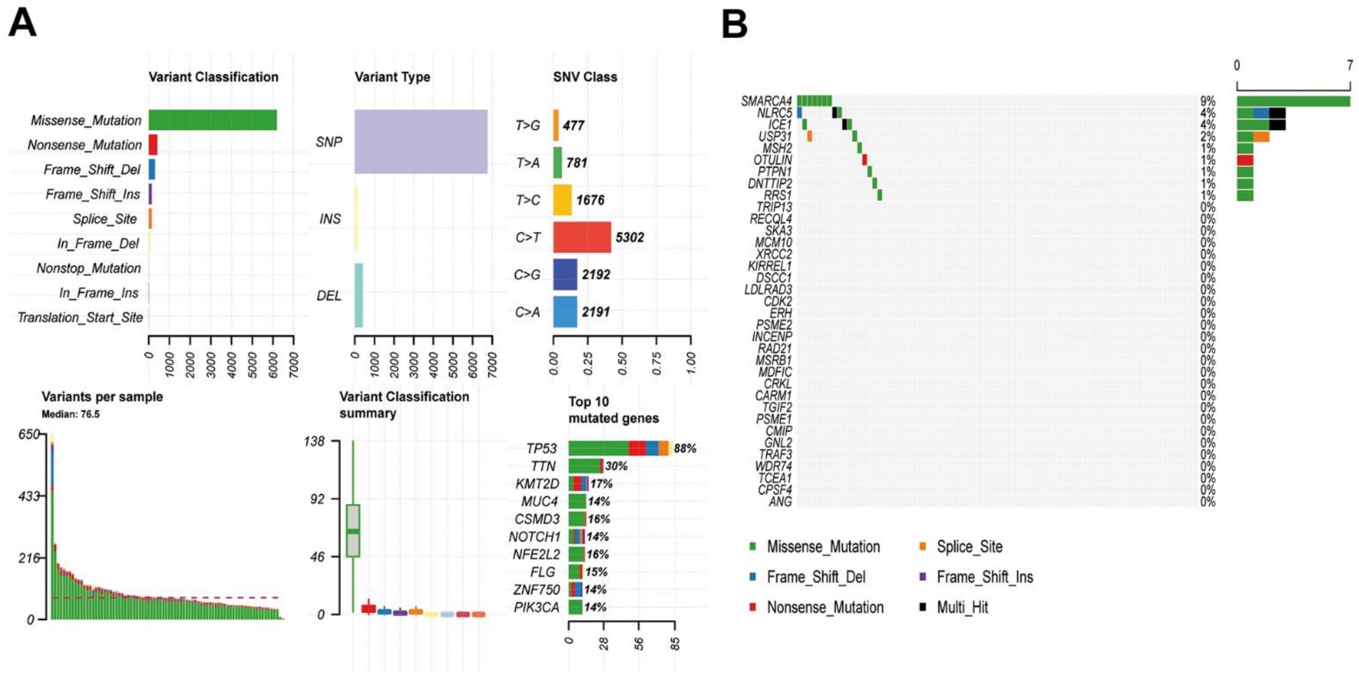

Supplementary Figure 1. Display of mutation rates of prognostic gene related to GRTTK in the TCGA cohort. (**A**) Whole-genome variation in the TCGA-ESCC cohort. (**B**) Waterfall plots showing somatic mutation frequencies of 35 key GRTTK in the TCGA-ESCC cohort.

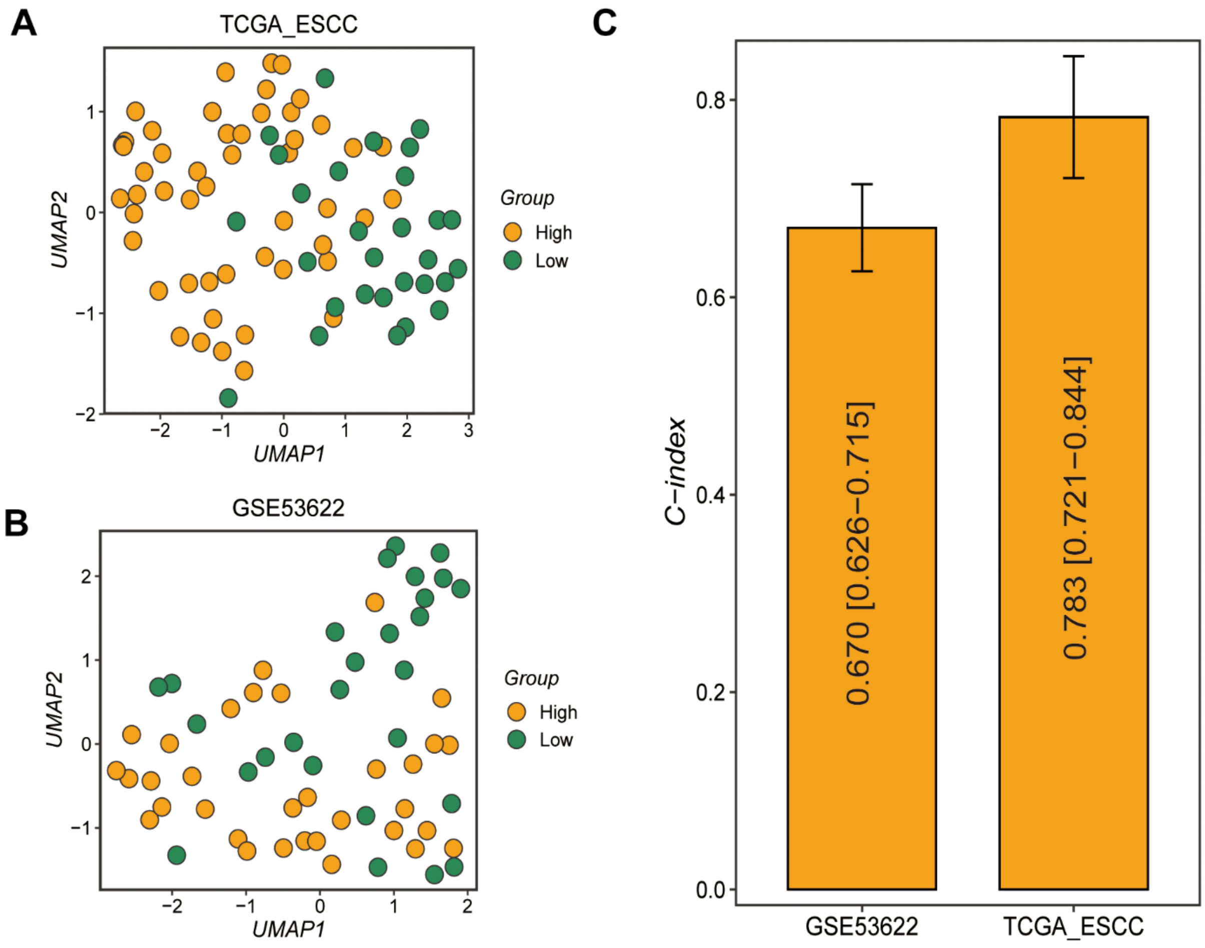

Supplementary Figure 2. UMAP analysis and C-index analysis in the training and validation dataset. (**A**) UMAP analysis reveals the distribution patterns of patients in the high and low-risk groups in TCGA-ESCC cohort. (**B**) UMAP analysis depicting the distribution patterns of patients in the high and low-risk groups in GSE53622 cohort. (**C**) The C-index was used to assess the performance of risk model.

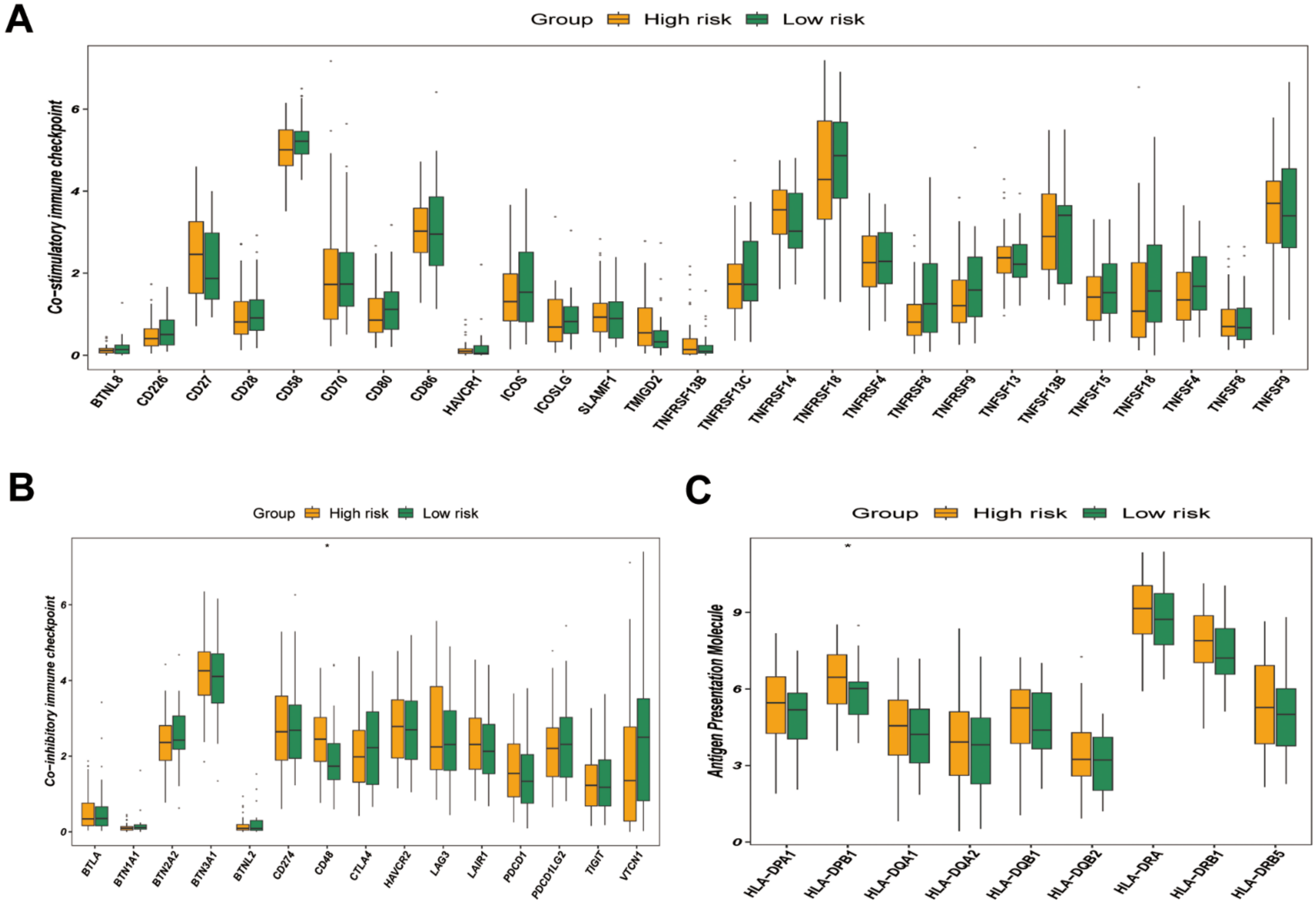

Supplementary Figure 3. Comparative analysis of multiple immune checkpoint molecules among different risk groups in the TCGA-ESCC cohort. (**A**) Costimulatory molecules expression distribution in the high-risk and low-risk groups of the TCGA-ESCC cohort. (**B**) Distribution of coinhibitory molecules expression in the high-risk and low-risk groups of the TCGA-ESCC cohort. (**C**) Expression profiles of antigen presentation molecule in the high and low-risk group of the TCGA-ESCC cohort.
