# Supplementary material for: Risk model based on genes regulating the response of tumor cells to T-cell-mediated killing in esophageal squamous cell carcinoma
Source: Aging (Albany NY). 2024 Feb 1;16(3):2494–516. doi: 10.18632/aging.205495 (PMC10911339; doi:10.18632/aging.205495)
Supplement: Supplementary Table 1 [file aging-16-205495-s002.pdf]

SUPPLEMENTARY TABLE

Supplementary Table 1. Univariate Cox regression analysis screened genes with similar prognostic value.

Genes	TCGA_ESCC	GSE53622
EIF3D	0.01467913	0.05363505
SPTLC2	0.07938117	0.06810316
EIF4H	0.01330395	0.02735845
RGP1	0.00032109	0.07588607
CDK2	0.04077468	0.08068879
TMEM209	0.08931848	0.04622017
CAPZA3	0.05082211	0.02880283
TCEA1	0.03995525	0.02700871
