# Supplementary material for: Risk model based on genes regulating the response of tumor cells to T-cell-mediated killing in esophageal squamous cell carcinoma
Source: Aging (Albany NY). 2024 Feb 1;16(3):2494–516. doi: 10.18632/aging.205495 (PMC10911339; doi:10.18632/aging.205495)
Supplement: Supplementary Table 3 [file aging-16-205495-s004.pdf]

Supplementary Table 3. Primers used for real-time PCR.

GENE	Forward (5'→3')	Reverse (5'→3')
CDK2	GACACGCTGCTGGATGTCA	CGTAGTGCAGCATTTGCGAT
TCEA1	CAAAGAAGCCATCAGAGAGCATCAG	TTGTCATTGGTTCATCAGCACTACG
TMEM209	CTCGCTACCGTTCTTCACCTACC	ACCCTATGCTGTTTCTCCTCTTCAC
GAPDH	GGAGCGAGATCCCTCCAAAAT	GGCTGTTGTCATACTTCTCATGG
